# Chemical Composition and Bioactivity Dataset Integration to Identify Antiproliferative Compounds in *Phyllanthus* Plants

**DOI:** 10.3390/pharmaceutics16111381

**Published:** 2024-10-27

**Authors:** Luis Diaz, Taylor H. Díaz-Herrera, Ericsson Coy-Barrera

**Affiliations:** 1 Bioprospecting Research Group, School of Engineering, Universidad de La Sabana, Chía 140013, Colombia; 2 Bioorganic Chemistry Laboratory, Universidad Militar Nueva Granada, Cajicá 250247, Colombia; u0500872@unimilitar.edu.co

**Keywords:** *Phyllanthus*, antiproliferative agents, lignans, bioactivity, covariate analysis

## Abstract

Background/Objectives: *Phyllanthus* species are renowned in traditional medicine for their diverse therapeutic properties, including potential anticancer activities. This study explored the antiproliferative potential of six *Phyllanthus* species by integrating chemical composition with bioactivity assays to identify key antiproliferative compounds. Methods: The integration of liquid chromatography–mass spectrometry (LC-MS)-based chemical composition data with antiproliferative activity against three cancer cell lines—PC-3 (prostate adenocarcinoma), SiHa (cervical carcinoma), and A549 (lung carcinoma)—as well as a normal mouse fibroblast line (L929) was performed by covariate analysis. These compounds were subsequently isolated and structurally characterized using spectroscopic methods. Results: Through covariate statistics, seven *m*/*z* features were found to be plausible active compounds, and after isolation, they were related to butyrolactone and arylnaphthalide lignans. Among the active isolates, an unreported compound, (+)-phyllanlathyrin **6**, was discovered in the aerial part of *Phyllanthus lathyroides*. The isolated compounds exhibited moderate to good antiproliferative activity (IC_50_ < 20 µM) with selectivity to SiHa, validating the covariate-based identification approach. Conclusions: These findings highlight the potential of *Phyllanthus* species as sources of novel anticancer agents, with specific arylnaphthalide lignans showing promising cytotoxic effects that could be further developed into therapeutic leads. Additionally, this study underscores the value of combining advanced analytical techniques with bioactivity testing to uncover bioactive compounds from natural sources. The results contribute to the growing body of evidence supporting the therapeutic relevance of *Phyllanthus* species and provide a foundation for future drug development efforts targeting cancer treatment.

## 1. Introduction

The genus *Phyllanthus* belongs to the family Phyllanthaceae and comprises over 2000 species distributed across tropical and subtropical regions of the world [1]. These plants have a long-standing history of use in traditional medicine, particularly in Asian, African, and South American cultures [2]. The therapeutic applications of *Phyllanthus* species are diverse, ranging from treating liver disorders, such as hepatitis and jaundice, to managing diabetes, urinary tract infections, and skin diseases [3]. Among these, *Phyllanthus niruri*, commonly known as “stonebreaker”, has gained significant attention due to its claimed benefits and pharmacological properties [4]. The ethnopharmacological relevance of *Phyllanthus* species can be attributed to their rich phytochemical profile, which includes a variety of bioactive compounds such as flavonoids, tannins, alkaloids, lignans, and polyphenols. These compounds have been linked to various biological activities, including anti-inflammatory, antioxidant, antiviral, and anticancer effects [5,6]. The growing interest in natural products as sources of novel therapeutic agents has led to an increased focus on *Phyllanthus* species as potential reservoirs of bioactive molecules, particularly in the context of cancer research [5].

The phytochemical composition of *Phyllanthus* species is notably diverse, encompassing a wide array of secondary metabolites that contribute to their therapeutic potential. Among these, lignans have emerged as a key group of compounds with significant bioactivity, particularly in terms of cytotoxic and antiproliferative effects [7]. Lignans are a class of polyphenolic compounds derived from the phenylpropanoid pathway, and they are characterized by a unique structure that consists of two phenylpropanoid units linked by a carbon–carbon bond [8]. These compounds are known for their ability to modulate various biological pathways, making them promising candidates for anticancer drug development [9]. Several lignans isolated from Phyllanthus species have demonstrated potent cytotoxic and antiproliferative activities against various cancer cell lines [5]. For instance, *Phyllanthus amarus* has been shown to contain lignans such as phyllanthin, hypophyllanthin, and niranthin, which exhibit cytotoxic effects against human colorectal cancer cells [10]. These lignans have been reported to induce apoptosis, inhibit cell proliferation, and disrupt cell cycle progression, thereby highlighting their potential as anticancer agents. In addition to their cytotoxic properties, lignans from *Phyllanthus* species also possess anti-inflammatory, antioxidant, and antiviral activities, further underscoring their therapeutic relevance [3,5,6].

Among the various lignan classes, butyrolactone, aryltetralin, and arylnaphthalene(ide) lignans stand out due to their remarkable bioactivity and structural diversity [7,11]. Butyrolactone lignans, such as (+)-hinokinin, are known for their ability to inhibit topoisomerase enzymes, which are essential for DNA replication and cell division [12]. This inhibition leads to the accumulation of DNA damage in cancer cells, ultimately triggering cell death. Aryltetralin lignans, including podophyllotoxin, have been extensively studied for their antiproliferative effects, particularly in leukemia and breast cancer [13,14]. These lignans exert their effects through multiple mechanisms, including the inhibition of microtubule polymerization, induction of apoptosis, and modulation of cell cycle regulators [14]. Arylnaphthalene and arylnaphthalide lignans, another important subclass, have also garnered attention due to their potent cytotoxic activity. These compounds are characterized by a naphthalene ring system alone or conjugated with a lactone moiety, respectively, and have been shown to induce apoptosis in various cancer cell lines through the activation of caspase enzymes and the downregulation of anti-apoptotic proteins [15,16]. The structural complexity and bioactivity of arylnaphthalene lignans make them attractive targets for developing novel anticancer agents. Exploring lignan bioactivity has expanded our understanding of their therapeutic potential and provided insights into the molecular mechanisms underlying their anticancer effects.

The search for effective anticancer compounds remains a critical focus in pharmaceutical research, as cancer continues to be one of the leading causes of mortality worldwide [17]. Natural products, particularly those derived from plants, have long been a cornerstone in drug discovery due to their structural diversity and biological activity. Many successful anticancer drugs, such as etoposide, paclitaxel, and vincristine, originate from natural sources, underscoring the potential of nature as a prolific source of novel therapeutics. The importance of anticancer compounds lies in their ability to selectively target and eliminate cancer cells, often through mechanisms that involve the induction of apoptosis, inhibition of cell proliferation, and disruption of critical cellular processes such as DNA replication and repair [18]. In the context of natural product-related pharmaceutics, integrating traditional knowledge with modern drug discovery techniques has led to the identification and development of promising new anticancer agents. These compounds offer potential efficacy and often exhibit reduced toxicity compared to synthetic drugs, making them attractive candidates for therapeutic development [19]. As resistance to existing cancer therapies grows, the continued exploration of natural products is essential to uncover new molecular frameworks that can be developed into next-generation anticancer drugs [20].

Given the promising cytotoxic and antiproliferative activities exhibited by bioactive lignans obtained from *Phyllanthus* species, the present study aims further to investigate the antiproliferative potential of six *Phyllanthus* plants. The study has been focused on integrating chemical composition data obtained through liquid chromatography–mass spectrometry (LC-MS) with bioactivity datasets to identify plausible active compounds responsible for the observed antiproliferative effects. The identification and isolation of these compounds have been undertaken to enable a more detailed estimation of their antiproliferative scope. The study encompasses a comprehensive analysis of the phytochemical profiles of the six selected *Phyllanthus* species, with particular emphasis on recognizing bioactive candidates. Thus, by correlating the LC-MS-based chemical composition data with the antiproliferative activity observed against cancer cell lines, the study aims to pinpoint specific compounds that contribute to the observed activity. The subsequent isolation and structural characterization of these compounds provided valuable insights into their potential as antiproliferative agents and laid the groundwork for future natural product-based drug development efforts.

## 2. Materials and Methods

### 2.1. General Information

All reagents (MTT, Folin-Ciocalteu, DPPH, ABTS, and TPTZ), standards (trolox, gallic acid, quercetin, podophyllotoxin, streptomycin, and penicillin), and solvents (EtOH, MeOH, CHCl_3_, CDCl_3_, and ACN) were commercially purchased from Merck KGaA and/or Sigma-Aldrich (Darmstadt, Germany) and were used without further purification. The purity of the dry solvents was ensured as specified upon purchase. Reaction progress and product purification were monitored using thin layer chromatography (TLC) on silica gel 60 F254 plates (Merck KGaA, Darmstadt, Germany), with detection under UV light at 254 nm.

### 2.2. Plant Samples

The aerial parts of six *Phyllanthus* plants were collected in different Colombian locations. In this regard, *P. urinaria* (4°0′40″ N; 73°46′56″ W, 457 m.a.s.l.), *P. caribaeus* (4°1′7″ N; 73°47′13″ W, 457 m.a.s.l.), and *P. caroliniensis* (4°0′40″ N; 73°46′56″ W, 457 m.a.s.l.) were collected in Acacías, Meta, whereas *P. salviifolius* (5°46′31″ N; 73°3′4″ W, 457 m.a.s.l.) was collected in in Duitama, Boyacá, and *P. madeirensis* (4°46′7″ N; 74°27′35″ W, 1800 m.a.s.l.) was collected in Anolaima, Cundinamarca. Finally, *P. lathyroides* (4°31′13″ N; 74°35′19″ W, 457 m.a.s.l.) was collected in Apulo, Cundinamarca. One sample of each species was deposited in the Colombian National Herbarium (Collection code: *P. urinaria* 596082; *P. caribaeus* 596079; *P. salviifolius* 596083; *P. caroliniensis* 596080; *P. madeirensis* 595478; *P. lathyroides* 596131).

### 2.3. Ethanolic Extracts Preparation

Healthy aerial parts (200 g) from the six *Phyllanthus* plants were air-dried, crushed, and ground. The ground aerial parts were separated into five replicates and then extracted with 96% ethanol using an orbital shaker (Heidolph Instruments GmbH & Co. KG, Schwabach, Germany). The procedure spanned a week, with daily solvent removal and restoration with clean 96% ethanol. The resulting mixtures were then concentrated under reduced pressure at 40 °C using a rotary evaporator (IKA^®^ Werke GmbH & Co. KG, Staufen, Germany). The resulting crude extracts were dried and stored at −20 °C until further analyses.

### 2.4. Antiproliferative Assay

Human cancer cell lines—prostatic adenocarcinoma (PC-3, ATCC CRL-7934), lung adenocarcinoma (A549, ATCC CCL-185), and cervical carcinoma (SiHa, ATCC HTB-35)—along with normal mouse fibroblasts (L929, ATCC CRL-6364) were cultured as monolayers in Dulbecco’s Modified Eagle Medium (DMEM) supplemented with 10% (*v*/*v*) fetal bovine serum (FBS), 1% (*v*/*v*) penicillin, and 1% (*v*/*v*) streptomycin. These cultures were maintained at 37 °C in a humidified atmosphere with 5% CO_2_. The antiproliferative effects of *Phyllanthus*-derived extracts and isolated compounds were evaluated using a previously described method [21]. Cells were seeded at 5 × 10^3^/well in 96-well plates (100 µL) and incubated for 24 h. After incubation, the medium was replaced with 100 µL of serum-free medium containing varying concentrations of the treatments (0.5–250 µg/mL for extracts and 0.01–100 µg/mL for pure compounds). Each treatment was tested in triplicate. Controls included a PBS-containing medium as a blank, 1% (*w*/*v*) bovine serum albumin-amended medium as a negative control (100% survival), and podophyllotoxin (0.16–100 µg/mL) as a positive control. After 48 h of treatment, cell viability was determined by adding 10 µL of MTT (5 mg/mL) to each well, followed by a 3-h incubation under the same culture conditions. The resulting formazan crystals were dissolved in 100 µL of DMSO, and absorbance was measured at 570 nm using a Varioskan LUX 96-well plate reader (Thermo Fisher Scientific, Waltham, MA, USA). The antiproliferative effects were quantified as the half-maximal inhibitory concentration (IC_50_) in µg/mL for extracts and µM for pure compounds. Dose–response curves were used to determine IC_50_ values from non-linear regression using GraphPad 7.0 (GraphPad, San Diego, CA, USA).

### 2.5. Chemical Characterization of Test Extracts

#### 2.5.1. Total Phenolic and Flavonoid Contents

The total phenolic and flavonoid contents were evaluated in the *Phyllanthus*-derived extracts using a previously reported method [22]. Briefly, the total phenolics content (TPC) was quantified using the Folin–Ciocalteu method, with absorbance measured at 765 nm. Results were expressed using a gallic acid standard curve (m = 7.70 × 10^−2^; b = 1.37 × 10^−2^; R^2^ = 0.99). Total flavonoid content (TFC) was determined using the aluminum chloride (AlCl_3_) complex method, with absorbance measured at 420 nm, and the results were expressed using a quercetin standard curve (m = 9.27 × 10^−2^; b = 1.02 × 10^−2^; R^2^ = 0.99).

#### 2.5.2. Antioxidant Capacity

The antioxidant capacity of the test extracts was measured by the FRAP (ferric reducing antioxidant power), DPPH^•^ (2,2-diphenyl-1-picrylhydrazyl), and ABTS^•+^ (2,2′-azino-bis(3-ethylbenzothiazoline-6-sulphonic acid) radical scavenging assays, measuring final absorbance at λ = 593 nm, λ = 515 nm, and 734 nm, respectively, following the previously reported method [22]. The results of DPPH^•^ and ABTS^•+^ assays were expressed as IC_50_ values from inhibition percentages for each free radical using GraphPad Prism v7.0. The free radical inhibition percentage (RI) was calculated using Equation (1).

(1) $$\%RI=\frac{\left(AbsC-AbsT\right)}{AbsC}\times 100\%$$

where AbsC = control absorbance value, and AbsT = treatment absorbance value.

#### 2.5.3. High-Performance Liquid Chromatography Coupled with Mass Spectrometry

Chemical characterization of the test *Phyllanthus*-derived extracts was also recorded through liquid chromatography coupled with mass spectrometry (LC-MS) using a Shimadzu Prominence HPLC system (Shimadzu Corporation, Kyoto, Japan) and a micrOTOF-Q II mass spectrometer (Bruker, Billerica, MA, USA) detector equipped with a quadrupole–time-of-flight (QToF) analyzer and electrospray ionization (ESI). Each aerial part-derived extract replicate (*n* = 5) was dissolved in absolute ethanol at a 5 mg/mL concentration, and a 20 µL aliquot was injected into the HPLC system. Separation was achieved on a Luna C18 column (4.6 mm × 150 mm, 5 µm; Phenomenex, Torrance, CA, USA) using a gradient elution method with solvent A (1% formic acid in Milli-Q H_2_O) and solvent B (1% formic acid in acetonitrile). The elution gradient was set as follows: 5% B (0–2 min), 5% to 30% B (2–7 min), 30% B (7–10 min), 40% to 90% B (10–22 min), 90% B (22–26) min, and returning to 5% B (26–30) min. The flow rate was maintained at 0.7 mL/min, and detection was monitored at a wavelength of 270 nm. Mass spectra were simultaneously recorded using positive ion mode electrospray ionization, scanning a mass range of 100–2000 *m*/*z*. The mass spectrometry parameters included quadrupole energy of 7.0 eV, collision energy of 14 eV, a curved desolvation line temperature of 250 °C, a heat block temperature of 400 °C, drying gas of 8 L/min, and a nebulization gas flow rate of 1.5 L/min.

### 2.6. LC-MS-Derived Metabolite Profile Pre-Processing

Mass spectrometry-derived data were processed using MZmine 2.53 [23]. The parameters used for processing were as follows: mass detection was set to centroid mode with an MS1 noise level of 5 × 10^2^. For the automated data analysis pipeline (ADAP) chromatogram builder, the settings included a minimum group size of 5 scans, a group intensity threshold of 4 × 10^2^, a minimum highest intensity of 4 × 10^2^, and an *m*/*z* tolerance of 0.01. Chromatogram deconvolution parameters were set with a minimum peak height of 4 × 10^2^, a peak duration of 0–2 min, and a baseline level of 4 × 10^2^. Isotope grouping was performed with an *m*/*z* tolerance of 0.01, a retention time tolerance of 0.1, and a maximum charge of 2. The joining aligner parameters included an *m*/*z* tolerance of 0.01, a weight for *m*/*z* of 70, a retention time tolerance of 0.1, and a weight for retention time of 30. Peak filtering was applied with a minimum peak in a row set to 5, an *m*/*z* range of 100–2000, and enabled the reset peak number ID option. Finally, gap filling was conducted with an intensity tolerance of 10%, an *m*/*z* tolerance of 0.01, and a retention time tolerance of 0.1.

### 2.7. Integration of Bioactivity and Chemical Composition Datasets

The pre-processed metabolite profile-derived data were saved as .csv files to create the feature intensity table (FIT), consisting of 2356 features across 30 samples. The resulting dataset was then autoscaled (unit variance scaling) for appropriate associations. MetaboAnalyst 5.0 (McGill University, Quebec, Canada) was used to generate an intuitive visualization of the distribution of autoscaled features [3]. The FIT was subsequently combined with the corresponding antiproliferative activity data, treated as a continuous variable, to form an integrated dataset. This matrix was then analyzed in SIMCA software (v 14.0) (Umetrics, Umeå, Sweden) for model construction using single-*Y* orthogonal partial least squares (OPLS), affording the respective scatter, variable importance in the projection (VIP) scores, and *S* plots.

### 2.8. Purification and Elucidation of Antiproliferative Candidates ***1***–***7***

Extracts from *P. lathyroides* (*Pl*) and *P. caribaeus* (*Pcb*) (500 mg each) were initially processed using solid-phase extraction (SPE) with Strata^®^ C18-U cartridges (55 µm, 70 Å, 500 mg, 6 mL; Phenomenex, Torrance, CA, USA). The cartridges were conditioned with methanol (6 mL) followed by water (6 mL) and methanol (6 mL). After loading the extracts previously dissolved in methanol, the cartridges were washed with water (5 mL) and then eluted with methanol (5 mL). The eluates containing the concentrated extracts were used for semi-preparative HPLC to isolate the target compounds.

Isolation was carried out on a UFLC Prominence system (Shimadzu, Columbia, MD, USA) in semi-preparative mode. This system included an LC-20AD pump, CTO-20AC column oven, SPD-20AV UV/Vis detector, SIL-10AP autosampler, and FRC-10A fraction collector, and was equipped with a reversed-phase Phenomenex Luna C18 column (250 × 10 mm, 5 μm; Phenomenex, Torrance, CA, USA) at 20 °C. The SPE-purified extracts (500 μL per injection, 80 mg/mL in MeOH) were injected ten times and separated at a flow rate of 3 mL/min using an isocratic elution method with solvents A (1% formic acid in H_2_O) and B (1% formic acid in ACN). Six targeted peaks were collected based on single-Y OPLS-based recognition, with the following retention times and yields: 6.0–6.2 min (3.6 mg, compound **1**), 11.5–11.7 min (11.3 mg, compound **2**), 12.6–12.8 min (11.4 mg, compound **3**), 13.9–14.0 min (8.7 mg, compound **4**), 18.9–11.1 min (5.4 mg, compound **5**), 19.6–19.8 min (4.9 mg, compound **6**), and 20.3–20.4 min (3.7 mg, compound **7**). The *Pl* extract yielded **1**, **3**, **6**, and **7**, while the *Pcb* extract yielded **2**, **4**, and **5**.

The structures of the isolated compounds were determined using ^1^H and ^13^C NMR spectroscopy, including the attached proton test (APT), on an Avance 400 spectrometer (Bruker, Billerica, MA, USA) with CDCl_3_ as the solvent (400 MHz for ^1^H; 100 MHz for ^13^C). All chemical shifts are reported in δ (ppm) with tetramethylsilane (TMS) as the internal standard. The APT ^13^C NMR data and optical rotation of the isolated compounds matched those reported for phyllanthusmin C (**1**) [24], (+)-acutissimalignan B (**2**) [25], phyllanthusmin A (**3**) [24], (−)-hinokinin (**4**) [26], justicidin A (**5**) [27], and justicidin P (**7**) [28]. Compound **6** was found to be an unreported compound, so its structural elucidation based on spectroscopic characteristics is presented herein.

(+)-Phyllanlathyrin (IUPAC: (+)-5-methoxy-9-(7-methoxybenzo[d][1,3]dioxol-5-yl)-8-oxo-6,8-dihydrofuro[3′,4′:6,7]naphtho[2,3-d][1,3]dioxol-6-yl acetate) (**6**): yellowish amorphous solid (mp 231–233 °C); [α]_D_^20^ = +48.9 (c = 0.02, CHCl_3_); ^1^H NMR (400 MHz, CDCl_3_) δ_H_ 7.76 (s, 1H), 7.58 (s, 1H), 7.33 (s, 1H), 6.86 (d, 1.4 Hz, 1H), 6.77 (d, J = 1.4 Hz, 1H), 6.06 (s, 2H), 5.92 (s, 2H), 4.03 (s, 3H), 3.86 (s, 3H), 2.18 (s, 3H). ^13^C NMR (100 MHz, CDCl_3_) δ_C_ 169.1 (C-9′), 167.5 (9-COCH_3_), 154.4 (C-3′), 151.8 (C-4), 150.8 (C-5), 150.1 (C-5′), 138.8 (C-4′), 136.7 (C-7), 135.2 (C-1′), 133.7 (C-1), 131.7 (C-2), 126.8 (C-8′), 124.6 (C-8), 123.3 (C-7′), 109.9 (C-2′), 109.2 (C-3), 104.5 (C-6′), 101.6 (3-OCH_2_O-5), 101.3 (3′-OCH_2_O-5′), 98.9 (C-6), 87.6 (C-9), 60.6 (7-OCH_3_), 56.5 (5′-OCH_3_), 20.4 (9-COCH_3_). HRESIMS (positive mode) *m*/*z* 467.0966 [M]^+^, (calcd. for C_24_H_19_O_10_, 467.0978).

### 2.9. Statistical Analysis

Standard deviations were calculated for the total flavonoid and phenolic contents, as well as for antioxidant capacities. IC_50_ values were determined using dose–response curves and regression analysis with GraphPad Prism 7.0 software. Normality was checked using the Shapiro–Wilk test. For normally distributed data, differences between treatments were analyzed with ANOVA followed by Tukey’s multiple comparison test. All statistical analyses were performed using InfoStat statistical software v29.09.2020 (National University of Córdoba, Córdoba, Argentina) (significance at α = 0.05).

## 3. Results and Discussion

### 3.1. Chemical Characterization: Total Flavonoid and Phenolics Content and Antioxidant Capacity

The phytochemical characterization showed the metabolite content at different levels in the six extracts as well as significance differences (*p* < 0.05) (Table 1). TPC values were highest in *P. salviifolius* (228.2 mg GAE/g DE), *P. caribaeus* (215.5 mg GAE/g DE), and *P. madeirensis* (215.2 mg GAE/g DE) extracts with respect to *P. urinaria* (173.1 mg GAE/g DE), *P. lathyroides* (142.6 mg GAE/g DE), and *P. caroliniensis* (139.8 mg GAE/g DE) extracts. Total flavonoids content displayed a similar behavior since *P. salviifolius* (17.3 mg QE/g DE), *P. caroliniensis* (15.9 mg QE/g DE), *P. urinaria* (14.2 mg QE/g DE), and *P. caribaeus* (13.3 mg QE/g DE) extracts showed the highest content with respect to *P. lathyroides* (9.2 mg QE/g DE) and *P. madeirensis* (9.2 mg QE/g DE) extracts. 

In addition, the five test extracts also showed antioxidant capacity by three assays at different levels (Table 1). The FRAP assay showed the highest antioxidant capacity for *P. madeirensis* (45.7 TEAC µM/mg DE), *P. salviifolius* (38.3 TEAC µM/mg DE), and *P. urinaria* (34.8 TEAC µM/mg DE) extracts. On the other hand, the antioxidant capacity for scavenging DPPH radical showed that *P. caribaeus* (IC_50_ = 17.6 µg/mL) and *P. urinaria* (IC_50_ = 19.9 µg/mL) extracts had the best IC_50_ values for this assay. In contrast, the ABTS assay showed the best IC_50_ for the *P. salviifolius* (IC_50_ = 7.3 µg/mL) and *P. caribaeus* (IC_50_ = 7.6 µg/mL) extracts (Table 1).

Antioxidant capacity and TPC and TFC have been reported in some *Phyllanthus* species. *P. amarus* has exhibited a phenolic content of 13.68 to 13.90 mg GAE/g DE and antioxidant capacity with DPPH radical scavenging activity (IC_50_ of 0.344 mg/mL) and FRAP (6.23 to 16.2 mg GAE/g sample) [29]. Liu et al. [30] and Bansal et al. [31,32] reported a phenolic content of 62 to 513 mg GAE/g extract and 250 to 262 mg GAE/L, respectively. Additionally, the antioxidant capacity for this species with IC_50_ values between 4.2 and 142 µg/mL has been measured. Fang et al. [33] reported the antioxidant capacity for isolated compounds from *P. urinaria* with IC_50_ values ca. 20 µM.

### 3.2. Characterization of Phyllanthus Aerial Part-Derived Extracts Based on LC-ESI-MS Data

**Figure 1:**
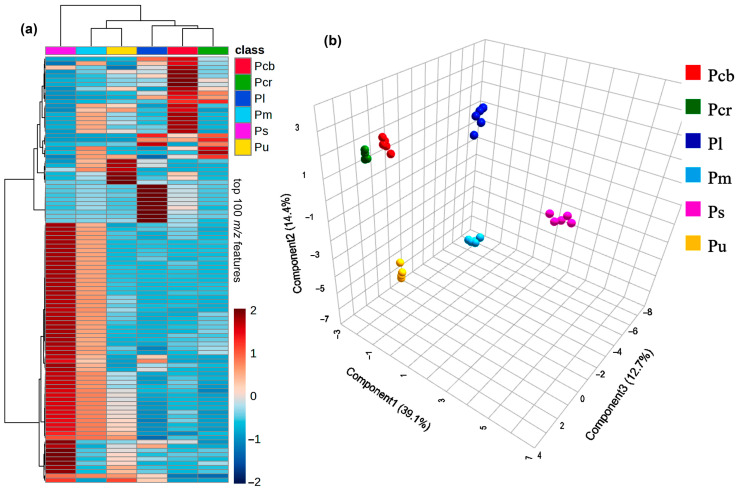
LC-MS-based differentiation of *Phyllanthus* aerial part-derived extracts from six plants and five technical replicates per plant. *Pcb* = *P. caribaeus*; *Pcr* = *P. caroliniensis*; *Pl* = *P. lathyroides*; *Pm = P. madeirensis*; *Ps = P. salviifolius*; *Pu = P. urinaria*. (**a**) Heat map depicting the chemical distribution based on the intensity of the top 100 most contrasting *m*/*z* features detected across *Phyllanthus* aerial part-derived extracts. Each column represents the average levels (*n* = 5) per plant extract, and each colored cell corresponds to the autoscaled intensity of a detected *m/z* feature, with the color scale ranging from dark red (high intensity) to dark blue (low intensity). (**b**) Three-dimensional score plot derived from sparse partial least square discriminant analysis (sPLS-DA), showing the separation of samples based on the three first principal components. Plant extract grouping was used as a categorical variable, explaining 66.2% of the variance.

A reverse phase LC-ESI-MS was employed to determine the chemical composition of the six extracts derived from *Phyllanthus* species. The mass-to-charge features recorded for each extract were extracted from the MS raw data and compiled into a feature intensity table (FIT), identifying 414 distinct features. This finding indicated a relevant metabolite diversity among the test extracts. While some features were shared across multiple extracts, others were unique to specific ones. This diversity was further visualized through a heat map illustrating a global LC-MS-based metabolite distribution using the top 100 differential metabolites according to the VIP scores (Figure 1).

The extracts were grouped into *Phyllanthus* species (*n* = 6) and the feature intensities were autoscaled to highlight differential metabolites. The heat map, with a color scale ranging from dark red (=2), indicating high feature intensity, to dark blue (=−2), indicating low feature intensity, revealed distinct metabolic profiles of the *Phyllanthus*-derived extracts (Figure 1a). In addition, a hierarchical clustering analysis (HCA) of the autoscaled data showed that the presence or abundance of specific metabolites clearly distinguished the *Phyllanthus* extracts. *P. salviifolius* was separated from the rest of the *Phyllanthus* plants, exhibiting a higher number of differential metabolites, possibly by the presence of flavonoids and other phenolics, which are very common in *Phyllanthus* plants [3]. The other five plants exhibited profiles that share components but were differentiated by the presence of particular metabolites, as expected, and deserve further investigation in future studies.

Finally, a sparse partial least squares discriminant analysis (sPLS-DA) further highlighted the chemical differentiation between the *Phyllanthus* chemical composition, with the first three principal components (PC1, PC2, and PC3) explaining 66.2% of the variance. The separation between the species was evident in the three-dimensional score plot (PC1 × PC2 × PC3) (Figure 1b), involving discrimination between *P. urinaria*, *P. salviifolius*, and *P. madeirensis* from *P. caribaeus*, *P. caroliniensis*, and *P. lathyroides* along C2, while *P. lathyroides* was separated from *P. caribaeus* and *P. caroliniensis* along C3.

### 3.3. Antiproliferative Activity of Phyllanthus Aerial Part-Derived Extracts

Several previous studies have explored the bioactivity of *Phyllanthus* plants against cancer cell lines [3,5]. However, *P. urinaria* is the only plant assessed for anticancer properties among the tested extracts. The antiproliferative activity of ethanolic aerial part extracts from the six *Phyllanthus* plants was evaluated against three cancer cell lines—PC-3, SiHa, and A549—as well as a normal cell line, L929. The results in Table 2 showed that the extracts exhibited varying degrees of antiproliferative activity, suggesting differential content and properties of antiproliferative compounds across the extracts.

The IC_50_ values ranged from 22.1 to 250 µg/mL. Notably, the extracts exhibited low activity against A549 (IC_50_ > 96 µg/mL) and showed lower activity against fibroblasts (IC_50_ > 207 µg/mL). In contrast, SiHa cells were the most sensitive cell line to the extracts, with an average IC_50_ of 74.3 µg/mL–except *P. madeirensis* (IC_50_ > 250 µg/mL)–followed by PC-3 cells, which had an average IC_50_ of 123 µg/mL. The *P. lathyroides*-derived extract was the most potent, with an IC_50_ of 22.1 µg/mL against SiHa cells, and exhibited good selectivity as it showed low activity against fibroblasts (IC_50_ = 225 µg/mL). In this regard, tested extracts demonstrated the lowest activity against A549 and moderate-to-low activity against PC-3 cells compared to that exhibited against SiHA. This pattern indicated that *Phyllanthus* aerial part extracts exhibit selectivity towards PC-3 and, particularly, SiHa cancer cell lines.

### 3.4. Recognition of Antiproliferative Candidates from Phyllanthus Plants Using Chemical Composition and Bioactivity Dataset Integration

**Figure 2:**
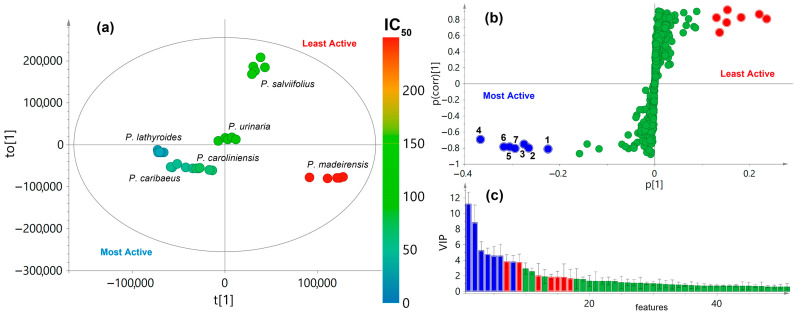
Integration of chemical and bioactivity datasets (i.e., feature-based chemical composition and antiproliferative activity) for *Phyllanthus* aerial part extracts using single-*Y* orthogonal partial least squares (OPLS) analysis. The IC_50_ values, represented as the continuous *Y*-variable, are depicted on a color scale (red = 250 µg/mL; aquamarine = 0 µg/mL). (**a**) Scores plot. (**b**) OPLS-derived *S*-plot. (**c**) OPLS-derived variable importance in the projection (VIP) plot.

The identification of active metabolites produced by *Phyllanthus* aerial parts was achieved by integrating antiproliferative activity and LC-MS-based chemical composition datasets through multiple-covariate statistical analysis. A single-Y orthogonal partial least squares (OPLS) modeling was employed to associate these datasets. Since the SiHa cell line was selectively affected by extracts according to the IC_50_ values (Table 1), it was utilized as the antiproliferative activity dataset, serving as the continuous Y variable. The OPLS regression incorporated t_1_ (a predictive score) and to_1_ (an orthogonal component). This incorporation effectively differentiated the aerial part extracts based on antiproliferative activity (Y-data) and chemical composition (X-data). This model demonstrated strong fit (R^2^X = 0.875, R^2^Y = 0.795) and predictability (Q^2^Y = 0.705), covering 51.3% of the variance in antiproliferative activity along t_1_ and 43.2% in chemical composition along to_1_. The discrimination mode among *Phyllanthus* aerial part extracts was revealed in the OPLS-derived scores plot (Figure 2a), where antiproliferative activity-influencing metabolite profiles were visualized through a color gradient, ranging from red (250 µg/mL) to aquamarine (0 µg/mL). The most active extracts (IC_50_ < 70 µg/mL) were grouped on the II and III quadrants, though involving low spreading, while the least active extracts were positioned on the I and IV quadrants with high variability among replicates (*n* = 5). This pattern advised that certain metabolites in those extracts with the highest activity would be associated with the resulting antiproliferative activity against the SiHa cell line.

To further pinpoint bioactive compounds, the OPLS-DA-derived loadings were analyzed using an *S*-plot conversion (p_1_ × p_(corr)1_), which categorized the statistical weight of differential compounds. The *S*-plot led to the visualization of the successful association of X-data with t_1_ through covariance and correlation under centering scaling (Figure 2b). This analysis highlighted the most significant metabolite distinctions between the least active (p1 > 0) and most active (p1 < 0) *Phyllanthus* aerial part-derived extracts, represented by those compounds distantly situated in the *S*-plot boundaries, indicating reliable metabolite discrimination. Hence, metabolite-based features **1**–**7** emerged as the maximum linking (p_(corr)1_ < −0.5, p1 < −0.2) with plausible bioactives, while the other seven features were strongly associated with the lowest bioactivity. The VIP plot (Figure 2c) confirmed the importance of these metabolites in the integrative discrimination process, identifying them as potential bioactive candidates (VIP scores > 3). These metabolites exhibited *m*/*z* and rt values in the ranges of 355.1165–513.1385 and 6.3–19.8 min, respectively, with specific rt/*m*/*z* pairs, pinpointed as 513.1385/6.3 (**1**), 357.1351/11.4 (**2**), 381.0984/12.3 (**3**), 355.1165/13.7 (**4**), 395.1144/18.2 (**5**), 467.0966/18.9 (**6**), and 425.1247/19.8 (**7**). Based on their LC and MS performance, these metabolite-related features were classified as compounds with middle polarity and low molecular weight. Feature **4** revealed the most substantial impact on the model due to its high classifying magnitude (p1 < −0.35) and was particularly abundant in *P. caribaeus* and *P. caroliniensis* extracts. Compounds 1 and **2** exhibited the highest reliability with their differential p(corr)1 values, indicating their presence across various most active *Phyllanthus* extracts. In this regard, all seven differential metabolites (**1**–**7**) were considered potential antiproliferative candidates, likely contributing to the observed activity against the SiHa cell line in the studied extracts.

This analysis demonstrated the successful application of single-Y OPLS for bioactive identification by integrating antiproliferative activity/chemical composition datasets. Principal component analysis (PCA) was not used as the primary multivariate statistical method since the use of supervised statistical methods like OPLS or PLS effectively maximized the covariate performance of differential features (independent variables) depending on antiproliferative activity (dependent variable), a task not as effectively achieved by unsupervised methods such as PCA [34]. Additionally, the fundamental benefit of associating datasets based on chemical composition lies in using LC-MS-based profiles as independent variables combined with the bioactivity of natural origin mixtures as the dependent variable [35]. Since single-Y OPLS requires a continuous variable, it is more convenient for multiple-covariate dataset integration than categorical variables, whose use can result in significant information loss [36,37]. Additionally, this method can detect unstable metabolites, which is crucial during extract fractionation [38]. However, a limitation of this approach is directing the analysis to false positives. This situation can be produced by other extract constituents synergistically or antagonistically acting against the target cell [39]. Therefore, the present study carefully performed the MS-guided isolation and studied their antiproliferative activity on cell lines of compounds **1**–**7** to validate the observed correlations.

### 3.5. Isolation and Identification of Antiproliferative Candidates

**Figure 3:**
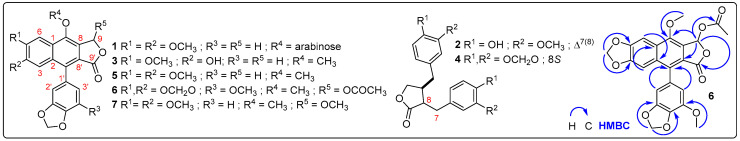
Structures of isolated compounds after statistical pattern recognition from *Phyllanthus* aerial parts. HMBC correlations in compound **6**.

Semi-preparative HPLC separations were carried out to purify compounds **1**–**7** from the tested active plant extracts (i.e., *P. lathyroides* and *P. caribaeus*). Compounds **1**, **3**, **6**, and **7** were isolated from the *P. lathyroides* extract, while compounds **2**, **4**, and **5** were obtained from the *P. caribaeus* extract. Once isolated, compounds **1**–**7** were identified and structurally elucidated through comprehensive NMR and MS spectroscopy analysis. These metabolites were identified as lignans, namely phyllanthusmin C (**1**) [24], (+)-acutissimalignan B (**2**) [25], phyllanthusmin A (**3**) [24], (−)-hinokinin (**4**) [26], justicidin A (**5**) [27], and justicidin P (**7**) [28], with their ^13^C NMR data corresponding to those reported in the literature. The structures of compounds **1**–**5** and **7** are presented in Figure 3. Contrarily, compound **6** did not match the previously reported data, so further elucidation was required. Hence, compound **6** was then characterized and structurally elucidated using various spectroscopic techniques. High-resolution mass spectrometry (HRMS) (Supplementary Material, Figure S1) established its molecular formula as [C_24_H_19_O_10_]^+^ with a quasimolecular ion peak at *m*/*z* 467.0966 [M+H]^+^ (error = 2.57 ppm). Its ^1^H NMR spectrum, recorded in CDCl_3_, revealed characteristic signals indicative of a polysubstituted polyaromatic moiety [40]. Specifically, the ^1^H NMR spectrum (Figure S2) displayed ten distinct singlet signals, and the ^13^C NMR spectrum presented twenty-four signals, which, upon detailed analysis using HMQC and APT experiments (Figure S3), corresponded to fourteen quaternary carbons, two methylene carbons, five methine carbons, and three methyl groups. Comparison of the ^1^H and ^13^C NMR data with literature values suggested a similarity to justicidin-like arylnaphthalide lignan [41], involving a very comparable NMR profile to that of justicidin P [28], isolated in this study as well. However, compound **6** differed from justicidin P by a singlet at δ_H_ 2.18 (3H), corresponding to a methyl group forming an acetyl group, and two doublets (*J* = 1.4 Hz) at δ_H_ 6.86 (1H) and 6.77 (1H) related to *meta*-positioned hydrogens attached to the aryl group, instead of a multiplet (3H) at 6.86–6.66 in justicidin P. A singlet at δ_H_ 6.08 (2H) was also attributed to a further methylenedioxy group at C4,C5. The aromatic region also exhibited signals characteristic of hydrogens forming an *ortho* system involving a tetrasubstituted aromatic ring, with two singlets at δ_H_ 7.76 (1H) and 7.58 (1H), corresponding to those protons at positions 3 and 6 of the arylnaphthalide lignan moiety. A singlet at δ_H_ 7.33 (1H) was assigned to the proton at position C9, geminal to the acetyl group. The ^13^C NMR spectrum provided additional structural details, including signals for aromatic carbons within the range of δ_C_ 98 to 155, consistent with the benzomethylenedioxy rings, signals for the methoxy groups at δ_C_ 60.6 and 56.5, and the carbonyl carbons at δ_C_ 169.5 (lactone) and 167.5 (acetyl). The HMQC experiment demonstrated the respective connectivity between hydrogens and their corresponding carbons and the HMBC experiment revealed heteronuclear long-range correlations, which were relevant in corroborating the above-mentioned structural assignments for **6** (Figure 3).

The hydrogens of the two methoxy groups correlated with the attached carbons in the arylnaphthalide moiety at δ_C_ 150.1 and 136.7, as well as the methylenedioxy-attached hydrogens, which correlated to the bearing carbons at δ_C_ 151.8/150.8 and 138.8/151.8, respectively. The signal at δ_H_ 7.33 (H-9) showed significant correlations with δ_C_ 136.7 (C-7), 167.5 (acetyl), 126.8 (C-8′), and 169.1 (C-9′), indicating the attachment of the lactone moiety to the naphthalene core structure. Additionally, correlations between H-2′ and H-6′ established the connection of the methoxymethylenedioxyaryl moiety to the naphthalide (Figure 3). In addition, compound **6** was found to be optically active, exhibiting a dextrorotatory orientation ([α]_D_^20^ = +48.9), likely due to the presence of a chiral carbon at C-9 bearing an acetyl group. However, determining the absolute configuration of this stereogenic center was not possible and will be addressed in a future study. Thus, compound **6** was identified as (+)-phyllanlathyrin (IUPAC: (+)-5-methoxy-9-(7-methoxybenzo[*d*][1,3]dioxol-5-yl)-8-oxo-6,8-dihydrofuro [3′,4′:6,7]naphtho [2,3-*d*][1,3]dioxol-6-yl acetate) (**6**), representing an unreported arylnaphthalide lignan isolated from *P. lathyroides* aerial parts.

The purified compounds **1**–**7** were further evaluated for their antiproliferative activity against the four cell lines to corroborate their OPLS-based bioactive differentiation. The resulting IC_50_ values are presented in Table 3. The confidence intervals (CI) for the IC_50_ values are also provided, indicating the reliability of these measurements. As anticipated, the SiHa cell line was the most susceptible to treatment with these compounds due to the focus on the LC-MS-based covariate pattern recognition, although some compounds exhibited good activity against PC-3 (i.e., **2** and **4**). Remarkably, lignans **1** and **6** demonstrated the most potent antiproliferative effect on SiHa cells, with an IC_50_ of 2.35 and 3.62 μM, respectively, although this outcome was less potent than the positive control, podophyllotoxin (IC_50_ = 1.85 µM). In addition, compound **1** exhibited moderate antiproliferative activity against PC-3 (IC_50_ = 18.3 μM) and SiHa (IC_50_ = 2.55 μM), with a notable decrease in efficacy against A549 (IC_50_ = 62.5 μM). It shows no significant cytotoxicity toward the fibroblast cell line L929c (IC_50_ > 100 μM). As mentioned, compound **2** was highly effective against PC-3 (IC_50_ = 6.55 μM) but much less so against SiHa (IC_50_ = 56.8 μM) and A549 (IC_50_ = 78.6 μM), with no observed cytotoxicity toward L929. Lignan **3** demonstrated balanced activity across all cancer cell lines, showing moderate IC_50_ values for PC-3 (16.3 μM) and SiHa (8.61 μM), though it was less effective against A549 (93.2 μM). It also exhibits some activity against L929 (IC_50_ = 92.3 μM). On the other hand, compound **4** showed potent activity against PC-3 (IC_50_ = 4.55 μM) and moderate activity against A549 (IC_50_ = 12.3 μM) but is less effective against SiHa (IC_50_ = 61.2 μM) and somewhat active against L929 (IC_50_ = 76.3 μM), while lignan **5** displayed moderate efficacy against PC-3 (IC_50_ = 17.6 μM) and A549 (IC_50_ = 50.3 μM), with better activity against SiHa (IC_50_ = 6.53 μM) and low activity against L929 (IC50 > 100 μM). Novel compound **6** demonstrated moderate activity against PC-3 (IC_50_ = 15.3 μM) and significant efficacy against SiHa (IC_50_ = 3.62 μM), with intermediate results for A549 (IC_50_ = 26.5 μM) and some activity against fibroblasts (IC_50_ = 86.3 μM). Similar to compound **6**, compound **7** showed moderate activity against PC-3 (IC_50_ = 15.2 μM) and SiHa (IC_50_ = 5.23 μM), with moderate results for A549 (IC_50_ = 45.3 μM) and the lowest activity against L929 (IC_50_ > 100 μM).

Hinokinin (**4**) is a well-known cytotoxic compound [42], and it is highlighted due to its strong activity against PC-3 and A549. Its moderate activity against L929 has suggested potential cytotoxicity concerns in non-cancerous cells. Similarly, (+)-acutissimalignan B (**2**) is another recognized cytotoxic lignan, previously isolated from the aerial part of *P. acutissima* [25], and it was highly selective for PC-3, with minimal activity against SiHa and A549, making it a possible lead compound for prostate cancer treatment with reduced off-target effects. Reports on the antiproliferative properties and mechanisms of action of (−)-hinokinin have been well-characterized. Cunha et al. [43] described a reduction in the G1 and S phases and an arrest in the G2/M phase, along with the downregulation of genes such as CCNA1 (Cyclin A1), CCND1 (Cyclin D1), and CCNE1 (Cyclin E1), and the upregulation of CDKN1A and CDKN1B (Cyclin Dependent Kinase Inhibitors 1A and 1B) in MFC-10A, MCF-7, and SKBR-3 cell lines. These findings suggest an action mechanism closely associated with cell division. Additionally, stimulation of apoptotic processes was observed, mediated by the expression of pro-apoptotic genes such as PUMA, NOXA, and the caspases CASP3 and CASP8.

Similarly, arylnaphthalene lignans, such as justicidin B, have demonstrated action mechanisms against proliferative cell lines. In glomerular mesangial cells (MCs), justicidin B exhibited inhibitory effects on factors involved in mitogen-induced processes, reduced cell proliferation induced by platelet-derived growth factor subunit B (PDGF-BB), and decreased levels of proliferating cell nuclear antigen (PCNA), which is associated with the S/G2 and G1 stages [44]. Noteworthy cell morphology changes, including elongation, shrinkage, and apoptosis, were observed, along with the downregulation of anti-apoptotic, apoptotic, and DNA repair proteins, such as Bcl-2, CASP3, and PARP-1 (poly(ADP-ribose) polymerase 1), in HeLa cells [45]. Furthermore, phytosterols like (-)-β-sitosterol-3-*O*-β-D-(6-*O*-palmitoyl) glucopyranoside, isolated from *P. songboiensis* extracts, have been shown to stimulate the production of IFN-γ in natural killer (NK) cells, contributing to the reduction of malignant cell transformation [46].

Phyllanthusmin C (**1**) and the novel (+)-phyllanlathyrin (**6**) show broad-spectrum activity across cancer cell lines, with less cytotoxicity toward fibroblasts. The secondary metabolites characterized in this study are likely involved in anti-apoptotic processes, the inhibition of DNA repair mechanisms, and/or the arrest of cell division processes. This fact makes them potential candidates for treating multiple cancer types. However, the positive control showed superior efficacy across all cell lines. This performance indicates that while the tested compounds **1**–**7** have potential, they require optimization to achieve comparable effectiveness. In this context, these findings provide valuable insights into the cytotoxic profiles of the covariate-based recognized lignans, highlighting compound **6** as a particularly promising candidate for further investigation. Future studies should focus on optimizing these compounds’ structures to enhance selectivity and reduce off-target effects. Additionally, the potential for combination therapies should be explored to maximize therapeutic efficacy. Additionally, it is essential to note that there may be other active compounds that were not detected due to potential antagonistic interactions among different extract components. Therefore, more in-depth integrative analyses are recommended to identify any missed bioactives, even those with lower activity but possible synergistic roles that could enhance the overall antiproliferative effect. Lastly, since various concentrations of compounds **1**–**7** were employed and they were distinct from those of the original extracts, the antiproliferative activity of the isolates cannot be compared with extracts. Consequently, further studies are needed to determine if **1**–**7** are the sole antiproliferative agents in the tested *P. lathyroides* or *P. caribaeus* aerial part-derived extracts.

## 4. Conclusions

The aerial part-derived extracts from the test plants were assessed for their antiproliferative effects against three cancer cell lines (PC-3, SiHa, and A549), demonstrating notable selectivity towards SiHa, with reduced toxicity to fibroblasts. This study represents the first investigation into the inhibitory potential of five *Phyllanthus*-derived extracts (excluding *P. urinaria*, previously reported) against these specific cancer cell lines, highlighting their preferential activity against SiHa. Additionally, integrating LC-MS-based chemical composition data with antiproliferative activity datasets provided a robust framework for identifying seven bioactive compounds in *Phyllanthus* species, underscoring the effectiveness of this approach in natural product research. Thus, the antiproliferative properties of these seven compounds, isolated from two *Phyllanthus* plants, validated the OPLS-based bioactive recognition, with compounds **1** and **6** showing the most potent activity against SiHa, indicating their potential as lead compounds for further development in cervical cancer therapies. Furthermore, this covariate-based integration led to the identification, isolation, and structural elucidation of a novel arylnaphthalide lignan, (+)-phyllanlathyrin (**6**), which exhibited selective activity against SiHa cells. However, our findings also allowed for the possibility of other active compounds being overlooked due to potential antagonistic effects within the extracts, suggesting the need for deeper integrative analyses. Overall, this study identified promising candidates for cancer treatment and emphasized the importance of further structural optimization and exploring combination therapies to enhance efficacy and reduce toxicity. These findings pave the way for future research aimed at developing novel anticancer agents from *Phyllanthus*-derived arylnaphthalene lignans.

## Figures and Tables

**Table 1:** Characterization of *Phyllanthus*-derived extracts.

Extract	TPC (mg GAE/g DE)	TFC (mg QE/g DE)	Antioxidant Capacity		
			FRAP (TE µM/mg DE)	DPPH (IC_50_ µg/mL)	ABTS (IC_50_ µg/mL)
*P. urinaria*	173.1 ± 20.6 ^b^	14.2 ± 0.2 ^c^	34.8 ± 0.9 ^b^	19.9 ± 1.7 ^ab^	8.5 ± 1.1 ^ab^
*P. caribaeus*	215.5 ± 5.9 ^a^	13.3 ± 0.7 ^c^	28.4 ± 0.2 ^c^	17.6 ± 1.6 ^a^	7.3 ± 0.7 ^a^
*P. salviifolius*	228.2 ± 5.4 ^a^	17.3 ± 0.1 ^a^	38.3 ± 2.7 ^b^	21.9 ± 4.6 ^ab^	7.6 ± 0.9 ^a^
*P. caroliniensis*	139.8 ± 6.2 ^c^	15.9 ± 0.3 ^b^	27.1 ± 0.5 ^c^	24.3 ± 1.7 ^b^	10.5 ± 0.8 ^b^
*P. lathyroides*	142.6 ± 5.6 ^c^	9.2 ± 0.4 ^d^	24.2 ± 0.7 ^d^	24.9 ± 1.5 ^b^	11.2 ± 0.7 ^b^
*P. madeirensis*	215.2 ± 6.1 ^a^	9.8 ± 0.5 ^d^	45.7 ± 1.1 ^a^	25.3 ± 1.2 ^b^	6.9 ± 0.6 ^a^

GAE = Gallic Acid Equivalent; QE = Quercetin Equivalent; DE = Dry Extract; TE = Trolox Equivalent; TPC = Total phenolics content; TFC = Total flavonoid content; FRAP = Ferric-reducing antioxidant power; DPPH = 2,2-diphenyl-1-picrylhydrazyl radical scavenging assay; ABTS = 2,2′-azino-*bis*(3-ethylbenzothiazoline-6-sulphonic acid radical scavenging assay. Values are expressed as mean ± standard deviation (SD), *n* = 3. Distinct lowercase superscript letters imply significant differences consistent with Tukey’s test (*p* < 0.05).

**Table 2:** Antiproliferative activity against cancer and normal cell lines of *Phyllanthus* aerial part-derived ethanolic extracts.

	PC-3 ^b^		SiHa ^b^		A549 ^b^		L929 ^c^	
Samples ^a^	IC_50_ ^c^	CI ^d^	IC_50_ ^c^	CI ^d^	IC_50_ ^c^	CI ^d^	IC_50_ ^c^	CI ^d^
*P. urinaria*	77.2	72.3–80.5	101	96.3–107	96.3	91.3–102	>250	-
*P. caribaeus*	112	107–117	49.8	45.7–52.8	188	179–196	235	227–244
*P. salviifolius*	95.3	90.7–101	131	126–135	133	122–139	207	201–213
*P. caroliniensis*	127	121–133	67.5	64.3–69.2	>250	-	>250	-
*P. lathyroides*	89.6	83.3–92.6	22.1	20.2–24.6	105	97.3–111	225	219–232
*P. madeirensis*	235	230–241	>250	-	>250	-	>250	-

^a^ Aerial part-derived extracts from *Phyllanthus* plants; ^b^ test human cancer cell lines: PC-3 (prostate adenocarcinoma), SiHa (cervical carcinoma), A549 (lung carcinoma); ^c^ normal cell line: L929 (murine fibroblasts), values expressed as half-maximal inhibitory concentration (IC_50_) in µg/mL; ^d^ CI = IC_50_ confidence interval (95% confidence) after non-linear regression.

**Table 3:** Antiproliferative activity against cancer cell lines and fibroblasts of compounds **1–7** isolated from *Phyllanthus* aerial part extracts.

	PC-3 ^b^		SiHa ^b^		A549 ^b^		L929 ^c^	
Compounds ^a^	IC_50_ ^c^	CI ^d^	IC_50_ ^c^	CI ^d^	IC_50_ ^c^	CI ^d^	IC_50_ ^c^	CI ^d^
**1**	18.3	17.3–19.2	2.55	2.46–2.67	62.5	60.2–64.1	>100	-
**2**	6.55	6.05–6.98	56.8	55.9–57.7	78.6	75.9–80.6	>100	-
**3**	16.3	15.5–17.3	8.61	8.21–8.77	93.2	90.6–96.1	92.3	90.0–94.4
**4**	4.55	4.38–4.66	61.2	58.9–63.5	12.3	11.5–13.0	76.3	74.9–77.2
**5**	17.6	16.8–18.2	6.53	6.01–6.89	50.3	47.8–52.6	>100	-
**6**	15.3	14.9–16.9	3.62	3.48–3.71	26.5	25.1–27.9	86.3	85.0–88.2
**7**	15.2	14.6–15.9	5.23	4.98–5.36	45.3	44.6–46.4	>100	-
**ppt ^e^**	2.22	2.03–2.37	1.85	1.78–1.96	4.82	4.62–5.13	2.53	2.44–2.61

^a^ Isolated compounds **1**–**7** from *Phyllathus* aerial parts (Figure 3); ^b^ test cancer cell lines: PC-3 (prostate adenocarcinoma), SiHa (cervical carcinoma), A549 (lung carcinoma)); ^c^ normal cell line: L929 (murine fibroblasts), values expressed as half-maximal inhibitory concentration (IC_50_) in µM; ^d^ CI = IC_50_ confidence interval (95% confidence) after non-linear regression. ^e^ ppt = podophyllotoxin used as positive control.

## Data Availability

Data are available from authors upon reasonable request.

## Supplementary Materials

The following supporting information can be downloaded at: https://www.mdpi.com/article/10.3390/pharmaceutics16111381/s1, Figure S1: HRMS spectrum of (+)-phyllanlathyrin (6); Figure S2: ^1^H NMR spectrum of (+)-phyllanlathyrin (**6**) (400 MHz, CDCl_3_); Figure S3: APT experiment of (+)-phyllanlathyrin (**6**) (100 MHz, CDCl_3_).
