# Supplementary material for: Chemical Composition and Bioactivity Dataset Integration to Identify Antiproliferative Compounds in Phyllanthus Plants
Source: Pharmaceutics. 2024 Oct 27;16(11):1381. doi: 10.3390/pharmaceutics16111381 (PMC11597697; doi:10.3390/pharmaceutics16111381)
Supplement: Supplementary file 1 [file pharmaceutics-16-01381-s001.zip › pharmaceutics-3191419-supplementary.pdf]

pharmaceutics

MDPI

Supplementary Materials: Chemical Composition and Bioactivity Dataset Integration to Identify Antiproliferative Compounds in *Phyllanthus* Plants

Luis Diaz, Taylor H. Díaz-Herrera and Ericsson Coy-Barrera

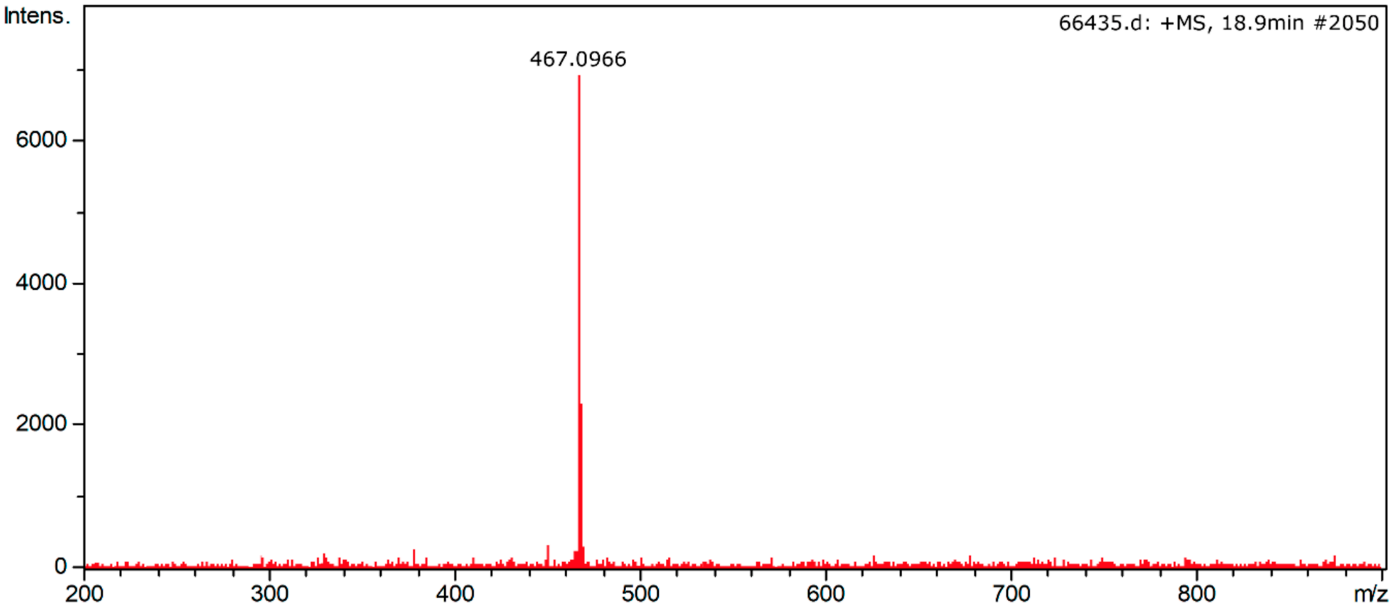

Figure S1. HRMS spectrum of (+)-phyllanlathyrin (6).

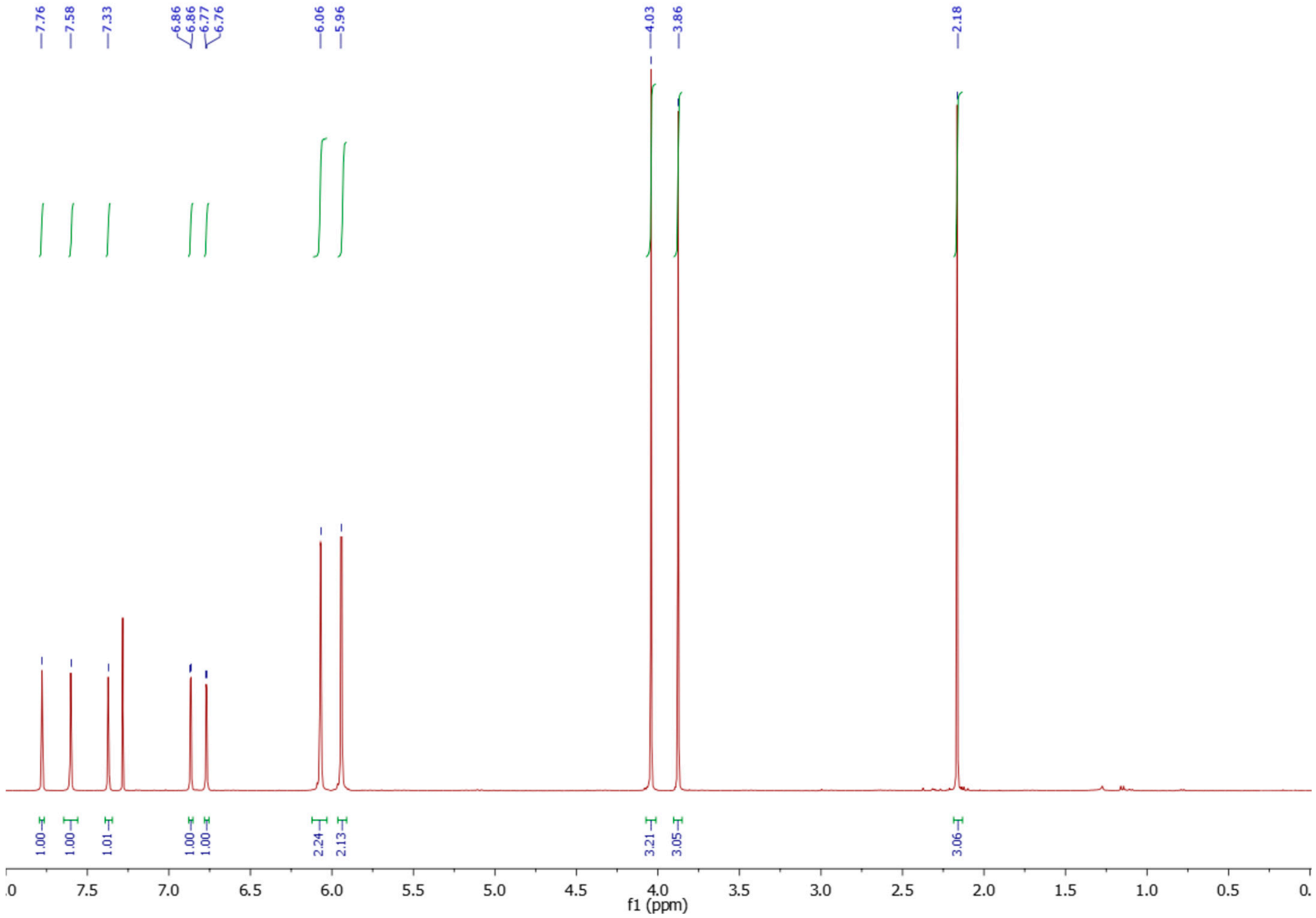

Figure S2. ^{1}H NMR spectrum of (+)-phyllanlathyrin (**6**) (400 MHz, $CDCl_3$).

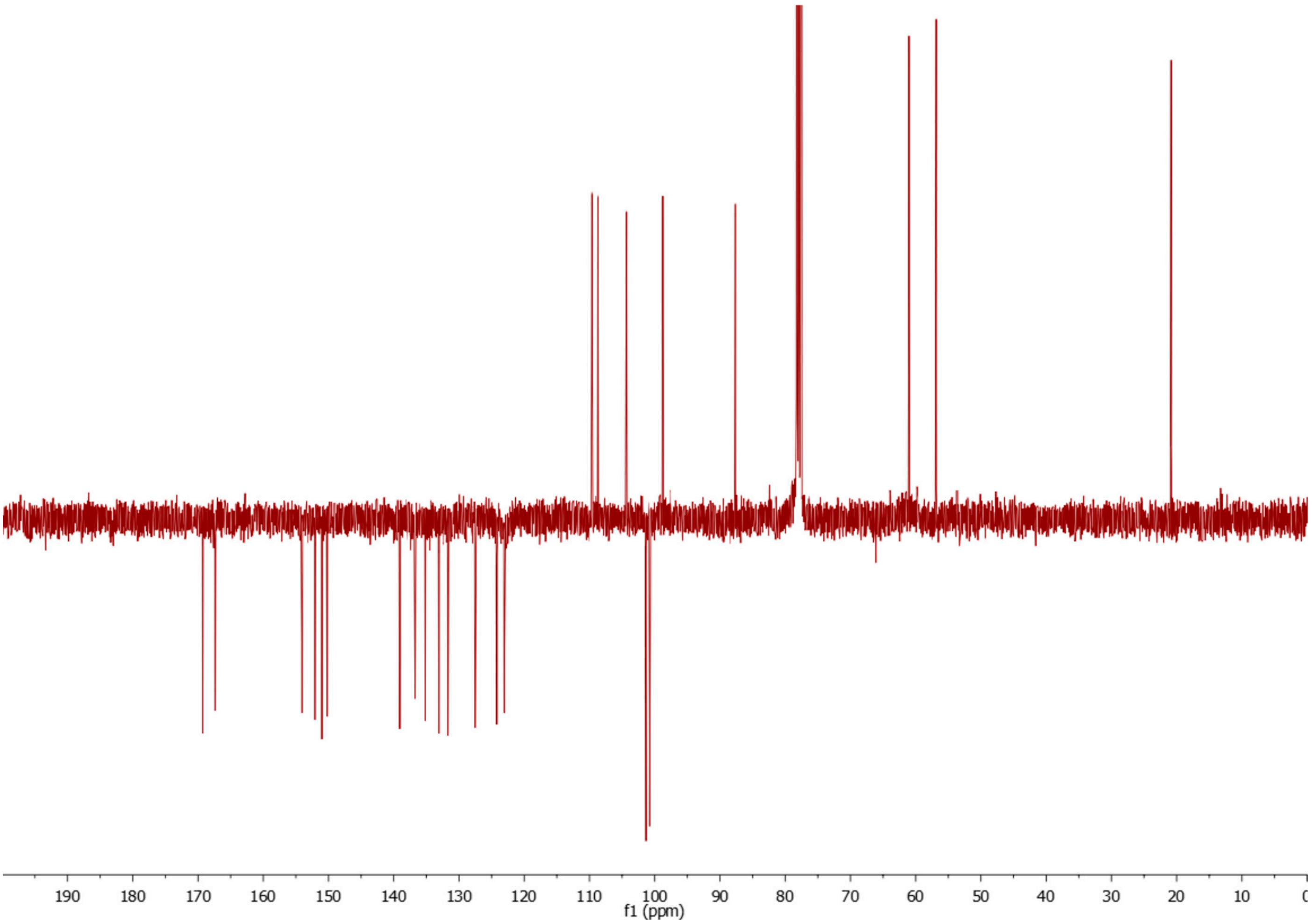

Figure S3. APT experiment of (+)-phyllanlathyrin (**6**) (100 MHz, $CDCl_3$).
